# Computation and Visualization of Regional-Scale Forest Disturbance and Associated Dissolved Nitrogen Export from Shenandoah National Park, Virginia

**DOI:** 10.1100/tsw.2001.452

**Published:** 2001-12-05

**Authors:** Keith N. Eshleman, Daniel A. Fiscus, Nancy M. Castro, James R. Webb, J Jr. Deviney

**Affiliations:** Appalachian Laboratory, University of Maryland Center for Environmental Science, Frostburg 21532, USA

**Keywords:** nitrogen, defoliation, gypsy moth, regionalization, Shenandoah National Park

## Abstract

Long-term watershed research conducted in Shenandoah National Park (SNP) in Virginia and elsewhere in the eastern U.S. indicates that annual export of dissolved nitrogen (N) from gaged forested watersheds to surface waters increases dramatically in response to vegetation disturbances. Dissolved N leakage is a common, well-documented response of small forested watersheds to logging in the larger region, while recent defoliation outbreaks of the gypsy moth (Lymantria dispar) larva in the deciduous forests of SNP have been shown to generate similar biogeochemical responses. A recent modeling analysis further suggests that a parsimonious, empirical, unit N export response function (UNERF) model can explain large percentages of the temporal variation in annual N export from a group of small gaged forested watersheds in the years following disturbance. The empirical UNERF modeling approach is completely analogous to the unit hydrograph technique for describing storm runoff, with the model representing annual N export as a linear deterministic process both in space and in time. The purposes of this analysis are to (1) test the applicability of the UNERF model using quarterly streamwater nitrate data from a group of ungaged watersheds in SNP; (2) demonstrate a park-wide application of a regional UNERF model that references the geographic distributions of bedrock geology and the timing and extent of gypsy moth defoliation over the entire SNP area; and (3) visualize the temporal and spatial patterns in vegetation disturbance and annual dissolved N export through the use of computer animation software. During water year 1992, the year of peak defoliation, our modeling study suggests that park-wide export had transiently increased by 1700% from a baseline rate of about 0.1 kg/ha/year. SNP forests appear to be characteristic of other N-limited second-growth forests in the eastern U.S. that leak little N under undisturbed conditions, despite receiving relatively large inputs of N from atmospheric deposition sources. Vegetation disturbances can apparently cause major changes in N input-output balances with potentially important ramifications for low-order forest streams and downstream receiving waters.

## INTRODUCTION

Long-term watershed studies conducted in Shenandoah National Park (SNP) in Virginia and elsewhere in the eastern U.S. indicate that annual export of dissolved nitrogen (N) from gaged forested watersheds to surface waters usually increases dramatically in response to vegetation disturbances. The leakage of dissolved N for several years following disturbance is a common, well-documented response of small forested watersheds to logging practices in the eastern U.S.[1]. Further, outbreaks of the gypsy moth (*Lymantria dispar*) larva, an exotic defoliator of eastern U.S. deciduous forests, have been shown to produce similar biogeochemical responses on oak (*Quercus* spp.) and mixed-oak watersheds in SNP[2,3]. N leakage from defoliated forests normally increases rapidly in the years following defoliation, peaking within a period of 1 to 3 years; a relatively long decay or recession in N leakage from the watersheds is then observed over an additional 4–8 years[3].

Interestingly, a modeling study of five intensively monitored forested watersheds in the Mid-Atlantic Highlands region (four located in SNP) suggested that multiple partial defoliations of forested watersheds produce rates of N leakage that are both proportional to the forested area defoliated and that are additive in time. A parsimonious, empirical linear systems model with these characteristics has been shown to explain large percentages of the temporal variation in annual N export from such defoliated watersheds in the years following disturbance. The model — known as the unit nitrogen export response function (UNERF) model — is completely analogous to the unit hydrograph method that is widely used for describing the characteristics of storm runoff attributable to one unit of excess precipitation onto a watershed. The UNERF model treats annual N export from disturbed forested watersheds as a linear deterministic process both in space and in time, with the specific model parameters estimated during the deconvolution process[4]:

$$N_w(t) = \int_0^t U(t-\tau) D_w(\tau) dt + B_w \tag{1}$$

where $N_w(t)$ is the N export from watershed $w$; $U(t-\tau)$ is a unit N export response function (UNERF); $D_w(\tau)$ is the proportion of forested watershed disturbed at time $\tau$ $(0 \leq D_w(\tau) \leq 1)$; and $B_w$ is the baseline N export from watershed $w$ in the absence of disturbance. The term "unit" in this case refers to the N export response to a complete disturbance of the watershed (i.e., when 100% of the watershed area is disturbed). The model thus includes two terms: (1) a convolution of a UNERF with the proportion of the watershed disturbed at time $\tau$, representing the integral response of the watershed to disturbance ($N_{w,D}$), and (2) a baseline N export response from the watershed without disturbance ($B_w$). The model represents the well-known response of a linear system to an "impulse" (in this case, a disturbance) and is governed by the principles of proportionality and superposition that allow the responses of the system to partial or multiple impulses to be predicted by convolution once the UNERF is known. Following the derivation of Chow et al.[5] of the discrete unit hydrograph, the N export response model was reformulated in the discrete time (annual) domain by incorporating a discrete convolution integral, representing the impulse response of the linear system:

$$N_{w,j} = \sum_{i=1}^{j \leq I} D_i U_{j-i+1} + B_w \tag{2}$$

where $N_{w,j}$ is the annual N export from watershed $w$ (kg/ha); $D_i$ is the proportion of the watershed disturbed in year $i$ (dimensionless); $U_{j-i+1}$ is a unit N export response function (kg/ha-yr); $B_w$ is the annual baseline N export from watershed $w$ in the absence of disturbance (kg/ha); $j$ is a counter for the number of annual time intervals; and $i$ is a counter for the number of disturbances ($I$ is the total number of disturbances). Eq. 2 indicates that the summation term is summed for $i = 1, 2, ..., j$ for $j \leq I$, but for $j > I$, the summation is limited to $i = 1, 2, ..., I$. Essentially, the modeling approach used to describe annual N export is analogous to the unit hydrograph method where the excess precipitation input has been replaced by a disturbance impulse and the unit hydrograph is replaced by the UNERF. In addition, a positive baseline N export is allowed (analogous to a base discharge in a unit hydrograph method which, for convenience, is ordinarily treated separately).

Since forest defoliation outbreaks frequently occur over relatively large regions of the landscape, it is appropriate to ask whether the spatiotemporal variations in N export from disturbed forested areas could be described using a regionalized version of the UNERF model. The purposes of the current study are to (1) test the applicability of a regionalized version of the UNERF model by making use of streamwater nitrate-N data for a group of ungaged watersheds in SNP; (2) demonstrate a park-wide application of a regional UNERF model that references the geographic distributions of bedrock geology and the timing and extent of gypsy moth defoliation over the entire SNP area; and (3) visualize the temporal and spatial patterns in vegetation disturbance and annual dissolved N export through the use of computer animation software.

## STUDY AREA

SNP was established in 1936 and traverses a 115-km segment of the Blue Ridge Mountains in north-central Virginia; ranging in width from 3 to 16 km, the park comprises nearly 800 km$^2$ of predominantly forested land, one of the largest contiguous areas of forest in the mid-Atlantic region (Fig. 1). By the time of the park's establishment, widespread logging and the chestnut blight had left SNP and most of the Appalachians devoid of virgin timber stands[6]. Forest succession in SNP following the elimination of the American chestnut (*Castanea dentata*) in the first half of the 20th century has been described as a "simple replacement" of chestnut by former associated species, such as the oaks, hickories, and yellow poplar, depending on topographic and edaphic factors[7]. These oak-dominated forests were heavily defoliated by the gypsy moth larva during the late 1980s and early 1990s. Climatically, the park is located in the humid subtropical zone; precipitation is uniformly distributed throughout the year, but is influenced appreciably by the park's topography. The geology of the park is dominated by three classes of rock: granite, basalt, and siliciclastic sedimentary rocks (Fig. 1); virtually all of the formations in the park have been subjected to significant metamorphic processes[6,8].

In a previous study, the UNERF model was parameterized for four intensively monitored forested watersheds in SNP (Fig. 1); each of these watersheds has been continuously gaged, has a long-term record of streamwater nitrate-N concentration and export data, and has a history of gypsy moth defoliation. An additional set of ten watersheds was not gaged, but has been regularly sampled (at least quarterly) for streamwater nitrate-N during the extended period when gypsy moth defoliation was occurring in SNP (Fig. 1). The set of 14 watersheds provides a useful diversity with respect to the geographic distribution of the three dominant rock types in the park (Fig. 1, insets), with several watersheds dominated by one lithological class and others comprised of substantial proportions of two or three classes.

## METHODS

Parameterized UNERF models for the four intensively monitored watersheds appear to vary as a function of the dominant lithological class. For example, the two siliciclastic watersheds (White Oak Run and Paine Run) show a relatively high peak in nitrate-N flux occurring 1 year after defoliation, while the basalt-dominated watershed (Piney River) shows an even higher peak that lags the defoliation by 2 years; the nitrate-N response of the granitic watershed (Staunton River) is highly attenuated and lags defoliation by 3 years (Fig. 2). The four parameterized models were used to develop composite lithology-based UNERF models for the three rock classes (Fig. 2, inset).

The lithology-based UNERF models were tested using field data from the ten ungaged watersheds. Assuming that the streamwater nitrate-N concentration measured under spring baseflow conditions is most representative of the annual discharge-weighted value, annual nitrate-N fluxes were computed for each watershed as the product of the spring nitrate-N concentration and an estimated mean annual runoff value of 0.50 m/year[6]; baseline annual nitrate-N export values ($B_w$) were estimated for each watershed using data collected prior to defoliation. Annual watershed defoliation (% area, 1986–1993) was calculated from digital maps obtained from SNP; these maps were based on sketch maps drawn during aerial overflights of the park during summer defoliation outbreaks (Dan Hurlbert, personal communication). Predictions of annual nitrate-N export from the ten watersheds were determined by linking the UNERF models to the lithology and defoliation data (30-m gridded layers) in a geographic information system (GIS, ArcInfo/ArcView); annual nitrate-N export from each grid cell was computed using the appropriate UNERF model and annual watershed export was

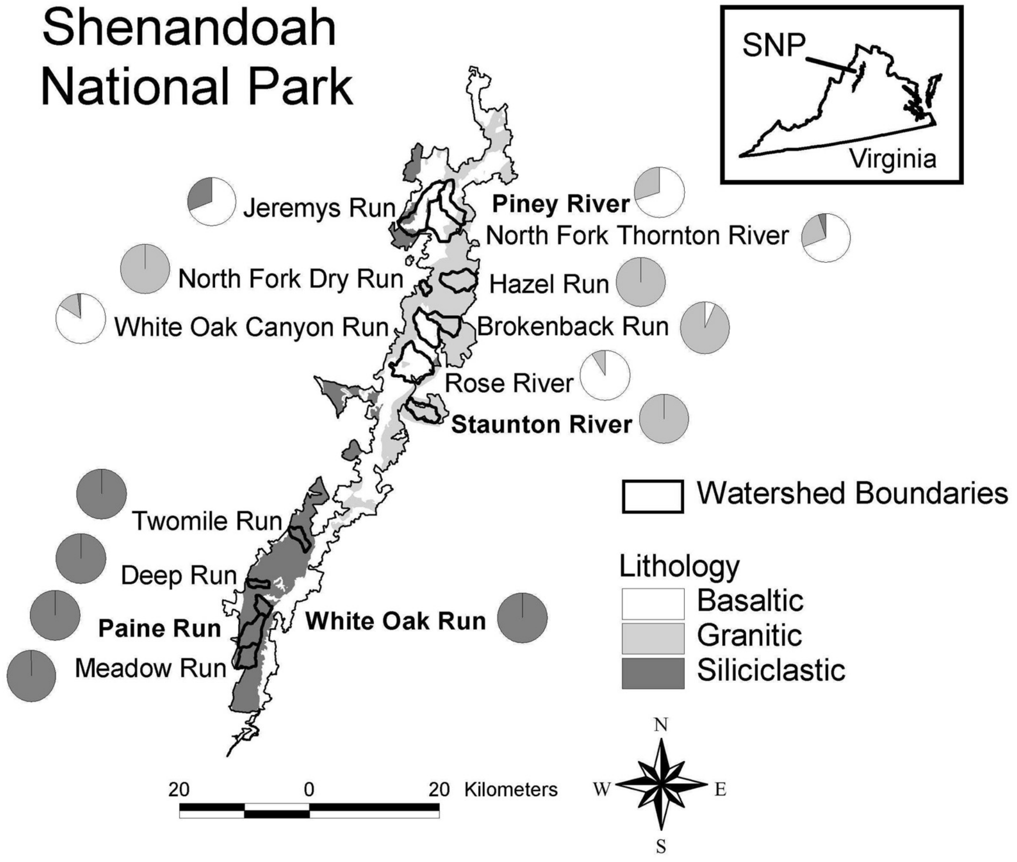

**FIGURE 1**. Geographic distribution of three lithologic classes in SNP in north-central Virginia; lithologic dominance within 14 watersheds (4 gaged watersheds shown in bold type) used in the study is also illustrated (insets).

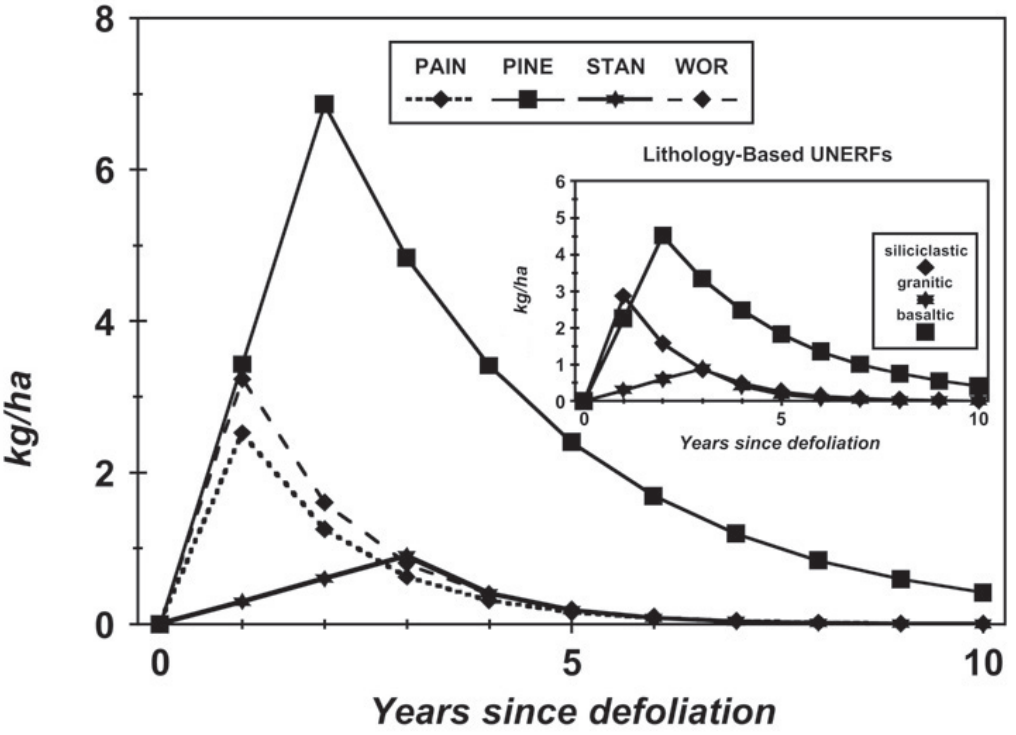

**FIGURE 2.** UNERFs for the four gaged SNP watersheds: Paine Run (PAIN), Piney River (PINE), Staunton River (STAN), and White Oak Run (WOR). Composite derived lithology-based UNERFs also shown (inset).

computed as the arithmetic mean of all grid cell values in a watershed. Following model testing for specific watersheds, we made park-wide estimates of annual nitrate-N export for the 20-year period from 1980–1999 using the same linked GIS and modeling approach; we set $B_w$ equal to 0.10 kg/ha/year, a value close to the mean value obtained for the ten ungaged watersheds. It was assumed that no gypsy moth defoliation occurred in SNP during the years prior to the first mapped defoliation in 1986, and that defoliation since 1993 has been negligible.

The GIS model operates on a cell-by-cell basis and on a yearly time step. The appropriate UNERF for the cell is determined by the lithology grid. If a particular cell is defoliated, an increase in annual N export is generated for all future years. Since N export is additive, the total N export from a particular cell in a particular year could be contributed from defoliation events that occurred up to 14 years prior. After estimating annual N export values from each of the 8 years of defoliation data, we summed N export contributions to obtain total export for each cell for each year. Since the cells were identical in size, we calculated annual watershed and park N export for each year as the arithmetic mean of the individual cell values.

In order to visualize both the spatial and temporal dynamics of disturbance and N export processes, we created a simple computer animation. Annual defoliation is visualized on a left-hand panel, while annual N export is shown on a right-hand panel. A bar chart illustrating the year-by-year variations is accumulated in a lower panel. Images of defoliation and N export for each year were generated in ArcView; these images were combined into a single animation file that can be set to repeat indefinitely. Base maps with topography, lithology, and stream channels were overlaid to provide a spatial context for the animation.

## RESULTS AND DISCUSSION

Gypsy moth defoliation in SNP began in 1986 in the northern end of the park and gradually moved southward along the Blue Ridge in subsequent years. From 1988 through 1992, roughly 25% of the park's forests were mapped as heavily defoliated (greater than 60% of leaf area removed) each year, although the specific defoliated areas often varied dramatically from year to year. Widespread defoliation ended in 1993, but coarser data from the state of Virginia suggest some defoliation in SNP during 1994. It is also likely that isolated defoliation outbreaks have occurred in the park in other years (Fig. 3).

Comparisons of estimated and predicted annual nitrate-N yields for the ten ungaged watersheds are shown in Fig. 4, with additional summary statistics presented in Table 1; statistical comparisons were made using linear regression and a calculation of model efficiency, E ($-\infty \leq E \leq 1$)[9]. In general, there is reasonably good agreement between the estimated and predicted yields, with most of the relationships showing relatively high $r^2$ values. Only one watershed (Deep Run) had a y-intercept that was statistically different from zero, and only one watershed (Brokenback Run) had a slope that was not statistically different from zero. In addition, a significant high bias is apparent for all three watersheds dominated by siliciclastic bedrock (Deep Run, Meadow Run, and Twomile Run). The same three watersheds,

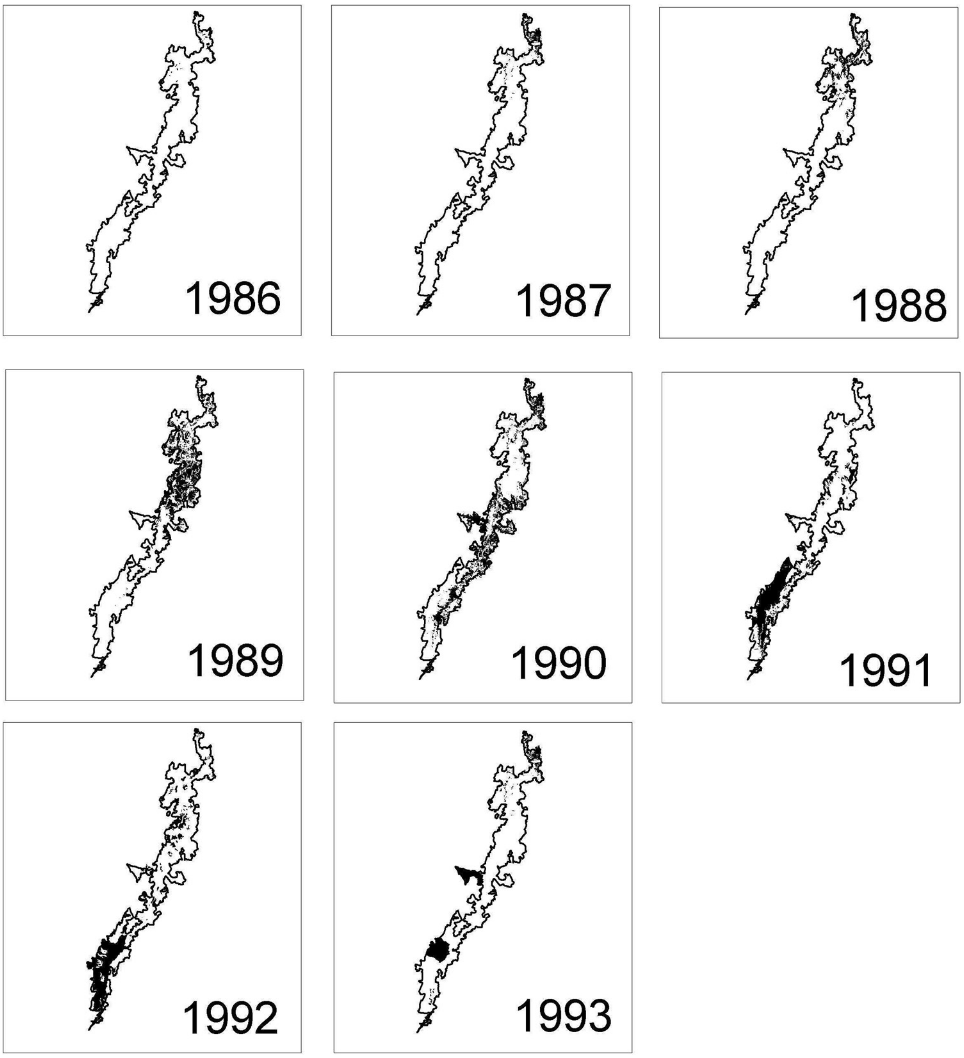

**FIGURE 3.** Geographical distribution of gypsy moth defoliation within SNP (1986–1993).

plus Brokenback Run and White Oak Canyon Run, also produced relationships with negative efficiencies (indicating that a mean value of the predictor would be a better model). Model predictions for the other five watersheds produced positive values of E.

Low model efficiencies (E values) do not necessarily reflect poor model performance, since the observations are only rough estimates of annual nitrate-N yield. Since discharge at one of the ungaged watersheds (Deep Run) had been calibrated against discharge at White Oak Run for several years, it was possible to produce a better estimate of nitrate-N yield using weekly streamwater nitrate-N concentrations and mean daily discharge data (i.e., the same computational method used by Eshleman et al.[3] for five gaged watersheds). Statistically, the relationship between predicted and observed annual nitrate-N yield is very similar to the relationship for Deep Run shown in Fig. 4, producing comparable values for the y-intercept, slope, $r^2$, and E (Table 1). This result suggests that the estimation procedure for annual nitrate-N export, based on an admittedly crude estimate of concentration and annual runoff, can apparently provide reasonable results. This is consistent with the interpretation that the lithology-based model may be overestimating nitrate-N yields from some defoliated SNP forests, particularly those underlain by siliciclastic rock.

Notwithstanding this overestimation problem, the modeling results largely verify the utility of the simplistic, lithology-based UNERF model. Since nitrate-N leakage from disturbed forested watersheds has been almost universally attributed to a combination of biological and hydrological factors[3,10,11,12], a predictive model based on lithologic classification was unexpected. Lithology, however, might indirectly predict N losses resulting from disturbance in an area like SNP, where there is a relatively tight coupling between bedrock, soil moisture, and forest species composition. In the southern Appalachians, the classic paper by Whittaker[13] pointed out the interaction between soil moisture conditions (as controlled by slope position and concavity) and elevation in determining vegetation in the Great Smoky Mountains. Compared to the southern Appalachians, the relatively steep dissected slopes — yet modest elevational range — of the Blue Ridge within SNP represent the type of setting where geological substrate rather than elevation may dominate the sec-

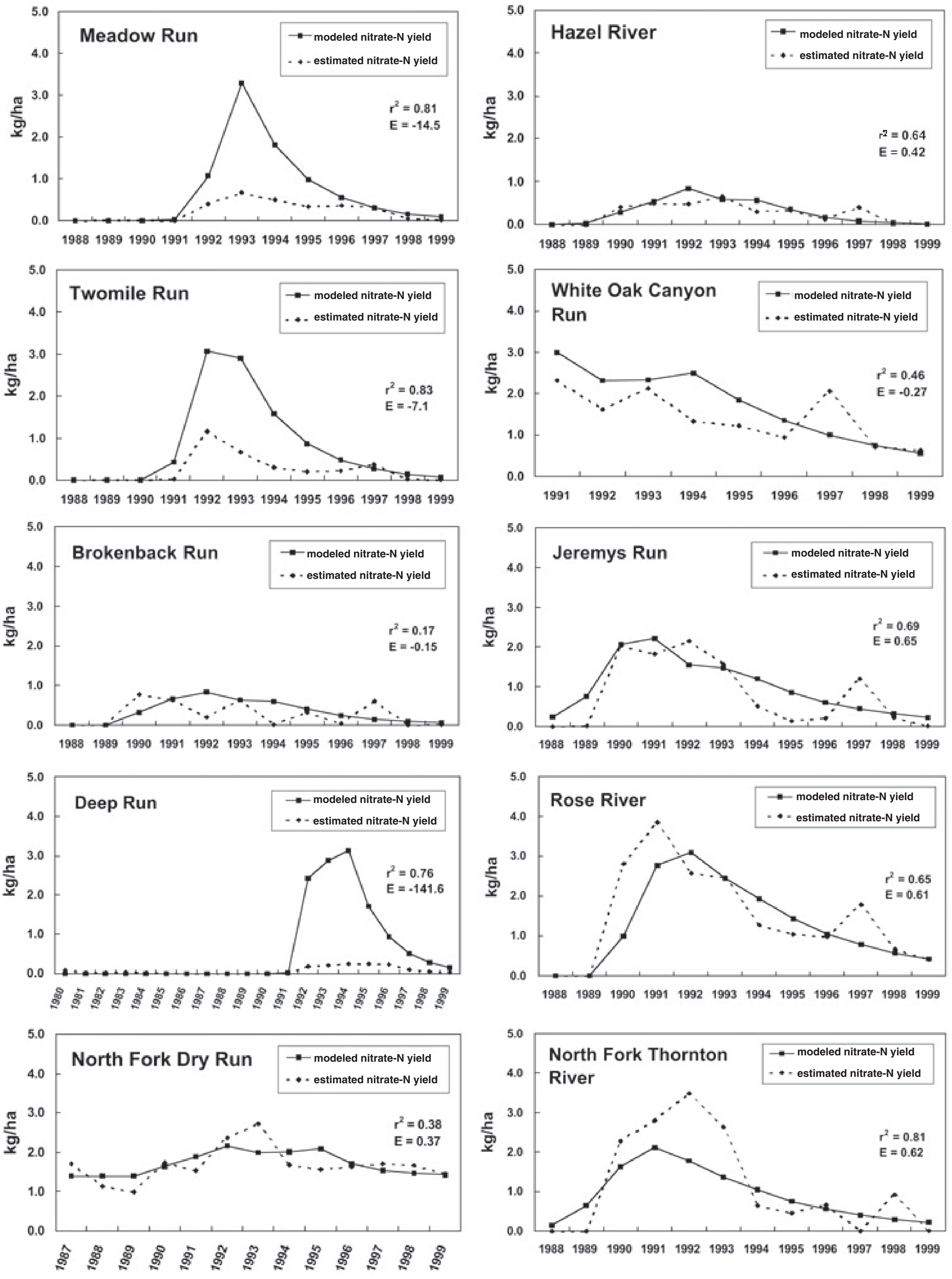

**FIGURE 4.** Graphical comparisons of predicted and estimated annual nitrate-N fluxes from ten ungaged SNP watersheds.

***Table 1. Statistical comparison of UNERF model predictions and estimated annual nitrate-N yields for ten ungaged SNP watersheds. Baseline nitrate-N yield assumed equal to 0.10 kg/ha-yr unless otherwise noted.***

| Watershed | Dominant Lithology | n | Intercept | Stat. sig. intercept?[1] | Slope | Stat. sig. slope?[2,3] | $r^2$ | E |
|---|---|---|---|---|---|---|---|---|
| Deep Run (est.) | S[4] | 20 | 0.03 | Y | 0.08 | Y, Y | 0.76 | -141.59 |
| Meadow Run | S | 12 | 0.07 | N | 0.21 | Y, Y | 0.81 | -14.49 |
| Twomile Run | S | 12 | 0.02 | N | 0.29 | Y, Y | 0.83 | -7.07 |
| N. Fork Dry Run* | G[5] | 13 | 0.07 | N | 0.95 | Y, N | 0.38 | 0.37 |
| Brokenback Run | G | 12 | 0.13 | N | 0.44 | N, N | 0.17 | -0.15 |
| Hazel River | G | 12 | 0.09 | N | 0.63 | Y, Y | 0.64 | 0.42 |
| Jeremys Run | B[6] | 12 | -0.20 | N | 1.03 | Y, N | 0.69 | 0.65 |
| N. Fork Thornton River | B | 12 | -0.44 | N | 1.74 | Y, Y | 0.81 | 0.62 |
| Rose River | B | 12 | 0.29 | N | 0.93 | Y, N | 0.65 | 0.61 |
| White Oak Canyon Run | B | 9 | 0.58 | N | 0.50 | Y, Y | 0.46 | -0.27 |
| Deep Run (comp.) | S | 17 | 0.03 | N | 0.13 | Y, Y | 0.74 | -44.27 |

*baseline export = 1.4 kg/ha-yr
[1]from zero @ $p < 0.05$
[2]from zero @ $p < 0.05$
[3]from unity @ $p < 0.05$
[4]S = siliciclastic
[5]G = granitic
[6]B = basaltic ondary forest succession process. In addition, it is well known that groundwater residence time and the occurrence of viable springs in the park are predicted largely by lithology. DeKay[14] describes the very large storages of groundwater that are common in the deep (3–20 m) regolith underlain by the basaltic rocks of the Catoctin Formation. In contrast, very few springs are observed in areas underlain by the metamorphosed sandstones and siltstones of the Antietam, Harpers, and Weverton Formations due to the extreme resistance to weathering of these siliciclastic rocks; these geological substrates promote rapid runoff and provide little storage of groundwater. Groundwater residence times and springs in the granitic Pedlar Formation are intermediate between the basaltic and siliciclastic bedrock as predicted by its intermediate resistance to weathering[6,14].

Therefore, we speculate that both forest vegetation and hydrological response of SNP watersheds are significantly — but indirectly — determined by substrate; it is these factors that largely control the rate and magnitude of the nitrate leakage response to forest defoliation. Forests on the siliciclastic rocks respond most quickly due to the short hydrologic residence time of these systems and also to the dominance on these sites of drought-tolerant chestnut oak (*Quercus prinus*), whose leaves are a preferred food of the gypsy moth larva. Forests on the basaltic rocks produce the greatest rate of nitrate-N leakage per "unit" defoliation, likely due to the much greater density of forest vegetation on these richer sites; the nitrate-N response of the basaltic watersheds lags the response of the siliciclastic watersheds, presumably due to longer hydrologic residence time. Finally, the nitrate-N responses of the granitic watersheds are the lowest per unit defoliation and the most attenuated, which can perhaps be explained by the high water-holding capacity of a deep saprolite that is known to exist in these deeply weathered systems.

Predicted park-wide estimates of annual nitrate-N export suggest a dramatic transient response to gypsy moth defoliation during the late 1980s and early 1990s (Fig. 5). The model predicts that park-wide annual average nitrate-N export from SNP forests increased exponentially from a rate of 0.1 kg/ha in 1986 to a rate of about 1.7 kg/ha in 1992. As defoliation declined in the early 1990s, the model suggests that average annual nitrate-N export exponentially declined from its 1992 peak to an estimated flux of about 0.2 kg/ha in 1999. Graphical displays of the predicted interannual spatiotemporal variations in nitrate-N export from SNP forests can be found at http://al.umces.edu/~fiscus/gypsy/Nexport_SNP.gif. A visualization of the predicted nitrate-N export from one of the ten ungaged watersheds can be found at http://al.umces.edu/~fiscus/gypsy/Nexport_NFThornton River.gif. The simple animations provide a general but integrated picture of the watershed disturbance model. Two aspects of the animations worth noting are (1) geology-driven spatial heterogeneity in the N export process within a watershed (and within

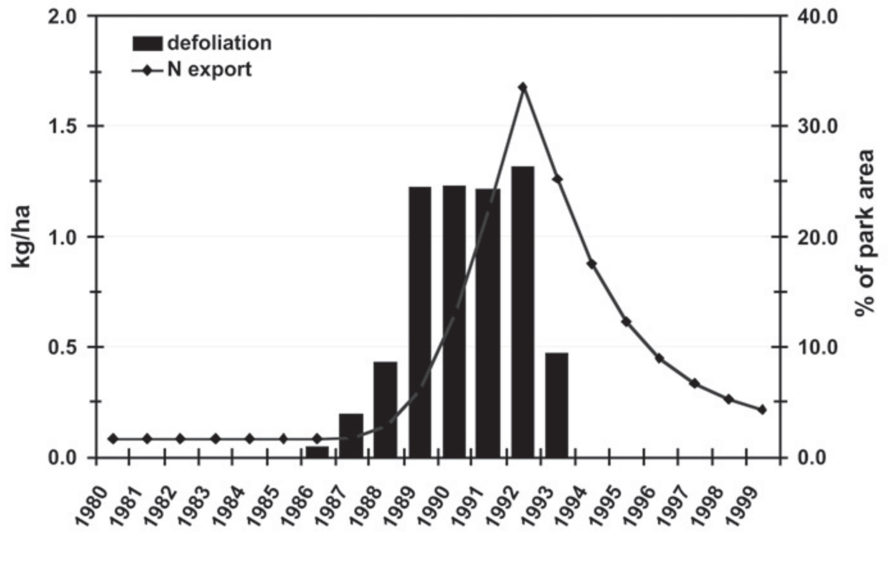

**FIGURE 5.** Gypsy moth defoliation and predicted annual nitrate-N export in SNP (water years 1980 through 1999).

SNP); and (2) the combined effect of this spatial heterogeneity, with additional spatiotemporal variation resulting from the interacting patterns of defoliation and the time-lag of the disturbance response. Given such spatial and temporal complexity, the ability of the UNERF to produce robust estimates is even more impressive.

## CONCLUSIONS

Gypsy moth defoliation of SNP forests during the late 1980s and early 1990s produced dramatic increases in annual nitrate-N yield from individual watersheds and from the park as a whole. These responses varied dramatically among 14 different watersheds in the park, but our model based on three dominant lithologic classes appears to adequately differentiate among the observed responses. During water year 1992 — the year of peak defoliation — our modeling study suggests that park-wide export had transiently increased by 1700% from a baseline rate of about 0.1 kg/ha/year. SNP forests appear to be characteristic of other N-limited second-growth forests in the eastern U.S. that leak relatively little N under undisturbed conditions, despite receiving relatively large inputs of N from atmospheric deposition sources. Vegetation disturbances can apparently cause major changes in N input-output balances with potentially important ramifications for low-order forest streams and downstream receiving waters.

## ACKNOWLEDGEMENTS

We thank Dan Hurlbert (Shenandoah National Park) for assistance in providing and interpreting the digital defoliation data for the study. M. Robbins Church and an anonymous referee provided constructive criticism of an earlier draft of the manuscript. We acknowledge support for the study through a STAR (Science to Achieve Results) program grant from the U.S. Environmental Protection Agency (EPA). Although the research described in this article has been funded in part by the EPA through grant CR826110-01-0 to the University of Maryland Center for Environmental Science, it has not been subjected to the Agency's required peer and policy review and therefore does not necessarily reflect the views of the Agency and no official endorsement should be inferred. This paper is scientific contribution 3505 from the University of Maryland Center for Environmental Science.

| | | |
|---|---|---|
| Received: | August | 9, 2001 |
| Revised: | November | 14, 2001 |
| Accepted: | November | 15, 2001 |
| Published: | December | 5, 2001 |
